# The significance of parental mentalizing for four-year-old children’s solitary pretend play

**DOI:** 10.1371/journal.pone.0297671

**Published:** 2024-01-31

**Authors:** Johanne Smith-Nielsen, Anne Christine Stuart, Katrine Isabella Wendelboe, Ida Egmose, Camilla Overbye Roos, Mette Skovgaard Væver

**Affiliations:** Department of Psychology, University of Copenhagen, Copenhagen, Denmark; Myanmar Health Network Organization, MYANMAR

## Abstract

**Background:**

Pretend play is a signature behavior of early childhood and is considered to reflect the child’s emerging symbolic function, enabling the interpretation of social signals, language development, and emotion understanding. While theory links parental mentalizing with children’s pretend play, only a few studies have investigated this association. These studies are limited to infancy and early toddlerhood, and child pretend play is assessed during play with an adult (social play). Based on the assumption that child solitary pretend play reflects the child’s ‘baseline’ pretend play ability, in this study, we investigated children’s pretend play at its peak, i.e., during the preschool age, without the facilitation of another player. The overall objective was to investigate if parental mentalizing increases pretend play complexity in children.

**Methods:**

The sample consisted 99 Danish mothers and their 4-year-old children. Employing a cross-sectional design, we hypothesized that parental mental state language, as an indicator of ‘online’ mentalizing during interaction with the child, is a mechanism through which ‘offline’ mentalizing, measured as parental reflective functioning, is associated with child solitary pretend play. Child pretend play complexity was observed and coded with an adapted version of the 12-Step Play Scale. Maternal offline mentalizing was assessed with the Parental Reflective Functioning Questionnaire, and maternal online mentalizing was assessed by coding the mothers’ mental state language during interaction with the child using a modified version of the mind-mindedness coding scheme.

**Results:**

While there was no direct effect of maternal offline reflective functioning on child pretend play, online mental state language mediated the link between offline maternal reflective functioning and child pretend play.

**Conclusions:**

These results provide support for the theoretically assumed link between parental mentalizing and children’s capacity for pretend play. Furthermore, our study contributes to the literature on parental mentalization, suggesting that parental mentalizing facilitates child development only if the parent can translate this ability into ’mentalizing in action’.

## Background

For decades, there has been an interest in understanding children’s pretend play as a predictor of various developmental outcomes, such as language, problem-solving, executive functioning, emotion regulation, and social skills (for a review, refer to Lillard et al., 2013 [1]). Less frequently, differences in children’s pretend play have been used as an outcome in themselves. Some researchers [2, 3] have suggested that pretend play should not only be considered as a phenomenon that affects other (more valued) developmental domains but studied in its own value. In the current study, we investigate the significance of parental mentalizing capacity for 4-year-old children’s pretend play.

### Pretend play in early childhood

Pretend play, also referred to as symbolic, fantasy, imaginary play [4], is a universal and cross-cultural signature behavior of early childhood [5, 6]. It is typically defined as the form of play where children use an object or language to serve as a ‘signifier’ (e.g., a block) to represent the meaning of another entity, the ‘signified’ (e.g., a cake) [7]. What is represented (pretended) during play can be any aspect of children’s lived or imagined experiences, including situations, activities, social roles, objects, or even the existing of nonexistent things. Pretend play typically begins around 12–18 months of age with object substitution (using one object to represent another) and typically peaks when children are between 3 and 5 years [8–10].

Pretend play has been studied as an individual phenomenon or process (solitary pretend play) [11] and as an interpersonal process between two play partners who co-create meaning and a shared reality (social play) [12, 13]. In pretend play, children often engage in role-playing, assuming different characters and enacting their actions, and dialogue within imagined scenarios. When participating in social pretend play, a play partner may introduce new perspectives, enhancing the complexity of the play. Conversely, during solitary pretend play, the child independently constructs the entire situation and may switch between roles while simulating conversations and interactions between the various characters involved in the play. This requires the ability to adopt another person’s perspective, imagining their thoughts, emotions, and actions and is regarded as a remarkable advance in children’s cognitive and social development [14, 15]. In the current investigation, we focus on children’s solitary pretend play, considering it as an indicator of their independent pretend play capacity without external scaffolding or facilitation from other players, whether they be children or adults.

### A window into the child’s emerging symbolic function

Researchers generally agree that playfulness indicates well-being and good mental health in children [5, 16, 17]. This is supported by numerous studies reporting that poor welfare (e.g., childhood depression, exposure to traumatic events, social deprivation, hunger, and non-optimal caregiving) is often accompanied by reduced playfulness and play abilities [18–21]. Yet, the function of pretend play in humans is being debated among play researchers [2, 22, 23], and the evidence for a unique function of pretend play for children’s development is inconclusive [1, 5].

While the answer to the question “why do children play?” remains unclear [5], theory and empirical studies consistently suggest that pretend play reveals the child’s emerging capacity for symbolic representation [4, 24]. Vygotsky proposed that during pretend play, the child develops an understanding that the use of objects can be separated from reality and their concrete representations of the world (e.g., a spoon can represent an airplane) and they learn that actions may be linked not only to the real properties of objects but also to the attribution of meanings in the imaginary situations [25]. In other words, the child creates a mental representation that is then projected onto objects, play behaviors, or situations [26, 27], and therefore the nature of pretend play may best be described as “as if play”: the child knows that these are not literal reflections of reality. Accordingly, researchers have proposed that a prerequisite for pretend play is the capacity for dual representation (real world and fantasy) and the ability to form meta-representations [27]. The player correctly perceives the real situation but, at the same time, intentionally represents the situation and objects differently. This capacity for symbolization (“representational skill”) is thought to underpin the interpretation of social signals, emotion understanding, and Theory of Mind (ToM: understanding that unobservable mental states, such as thoughts, beliefs, and emotions, underlie most human behavior [28]) as well as the interpretation of other symbols and language development [4, 5, 24, 27]. Support for this notion comes from a study in which Canonical correlation analysis revealed a construct (referred to by the authors as a “symbolic substrate”) underlying language, specific symbol understanding, and pretend play at 2–3 years, that predicted ToM at 4–5 years over and beyond pretend play itself [24]. Another study found that 3-5-year-olds’ propensity towards pretend play (conceptualized as ‘fantasy orientation’, reported by their preschool teachers) predicted their ability to differentiate between real and apparent emotion, and their ability to understand and appropriately engage in others’ emotions [29].

Individual differences in children’s capacity for pretend play, sometimes also referred to as play quality (e.g., [30]) or play sophistication (e.g., [12]), can be operationalized as the complexity of play [31]. Belsky and Most [32] developed a 12-Step Play Scale to assess the complexity of children’s play. In its simplest form, pretense behavior is directed towards the child itself (e.g., the child pretends to eat food from an empty plate) or towards an object (e.g., the child pretends that a teddy bear eats from an empty plate). Play complexity increases with object substitution where objects are imaginatively transformed (e.g., a stick becomes a horse, or a deck of cards becomes a phone), and the most complex form of pretend play is sequenced play where children construct elaborate narratives with multiple object substitutions. Individual differences in pretend play become more evident as the child develops, and the play complexity increases [31, 33]. In our study, we interpret higher levels of pretend play complexity as indicative of a more advanced symbolic representational ability in children.

### Parental mentalizing and children’s pretend play

The developmental context and caregiving environment havelong been assumed to be important for children’s pretend play abilities [34, 35]. Echoing this, Vygotsky (1967) described how the “more knowledgeable other” (e.g., a parent) facilitates pretend play and symbolic representation by offering alternative interpretations of reality [25, 35]. For example, during play, by representing a pen as an airplane; or during child distress, by interpreting the child’s behaviors as reflecting specific mental states (“Maybe you threw the pen away, because you are sad and don’t want the play to end?”) [25]. Studies consistently find that parents who actively participate in their child’s play activities (e.g., help them concentrate on the play and scaffold their behavior), in combination with showing positive affect, affectionate touch, and non-restrictive and non-intrusive behaviors, increase the child’s production of symbolic acts and pretend play complexity [13, 36–38]. What enables parents to interact with their child in such a manner? Firstly, it requires that the parent can take the child’s perspective, and secondly, that they can understand the ‘play topic’, recognize and envision the meaning of the child’s play behaviors; in other words, that the parent can *mentalize*. Mentalizing is a unique human ability that enables us to infer and interpret the behavior of others and ourselves as the result of underlying mental states (emotions, intentions, cognitions, and motives) [39].

In the context of the parent-child relationship, parental mentalizing is defined as the parent’s tendency to treat their child as a psychological agent with a mind of their own, that is, the parent’s willingness to guess and envision what mental states may lie behind the child’s immediate behavior [40, 41]. Theory and empirical findings suggest that parental mentalizing is vital for a range of child psychosocial outcomes, such as the development of emotion regulation and emotion understanding in self, and others (e.g.,[42–48]), executive functions, and language development [49]. It is assumed that mentalizing enables parents to provide a framework of presenting the child to internal experiences by “playing along” with the child’s mental world [50, 51] and that this process is fundamental for the child’s emerging understanding of the representational nature of the external world [40, 51]. Importantly, parental mentalizing not only entails a mental process of the parent (imagining the child’s feelings, motives, or cognitions), but also requires that the parent is able to translate this into ‘mentalization in action’, for example, by using language about mental states in the presence of the child in a coherent and meaningful manner [52, 53]. By this, the child comes to understand the relationship between mental representations (internal reality) and behavior (external reality), while at the same time comprehending that mental states are not equalized with the outer world. This process is very similar to, and assumed to be nourished in, pretend play [27, 54]. Based on the assumption that parents’ ability to mentalize or “play along” with the child’s inner world is a key ingredient of the caregiving environment that facilitates children’s pretend play, in this study, we investigate parental mentalizing as a predictor of children’s pretend play complexity.

### ‘Offline’ and ‘online’ parental mentalizing

Several conceptualizations and operationalizations of parental mentalizing exist [55]. Parental reflective functioning (PRF) is one construct widely applied in the parent-child literature and refers to parents’ ability to reflect on their child’s behavior, themselves as a parent, and the parent-child interactional dynamic [56]. PRF can be measured by coding of an interview (e.g., the Parent Development Interview: PDI; [56, 57]), and via self-report using a questionnaire (e.g., The Parental Reflective Functioning Questionnaire (PRFQ; [41, 58]). While both approaches assess parental mentalizing abilities ‘offline’, i.e., outside the immediate parent-child interactional context, other measures focus more specifically and explicitly on ‘online’ mentalizing abilities, i.e., parental mentalizing *during* parent-child interaction [55, 59]. Parents’ use of mental state language during parent-child interaction has been used to assess their propensity to put their own and their child’s minds into words during interaction [12, 42]. Although mental state language is not necessarily synonymous with the construct of mentalizing, it is nevertheless considered to indicate ‘mentalizing in action’ [52], or ‘online mentalizing’. While several methods to assess mental state language currently exist, parental mind-mindedness is one of the most widely used methods for examining parental mentalizing through verbal enactment [60, 61]. There are two ways of assessing mind-mindedness, each capturing different aspects of parental mentalizing: 1) Interactional (‘online’) mind-mindedness, which is an observational measure, where the parent’s comments on the child’s mental states are coded from parent-child interaction; and 2) Representational (‘offline’) mind-mindedness, which is coded on transcripts of a brief interview where the parent’s propensity to describe the child in terms of mental attributes are coded [60].

Since offline and online mentalizing refer to a similar mental activity (attending to mental states), it has been proposed that online mentalizing is the application of an underlying offline mentalizing capacity in a specific interactional context [51, 52]. Interestingly, however, only a few studies have examined associations between offline and online parental mentalizing. Links between online and offline mentalizing have mostly been examined using the mind-mindedness coding scheme, where some studies have shown moderate positive concurrent and longitudinal associations between representational (offline) and interactional (online) mind-mindedness among mothers of infants [62] and children aged 3–6 year [63]. In contrast, findings on links between offline measures of PRF and online mentalizing are inconsistent. Rosenblum and colleagues [64] reported that PRF (assessed in an interview) predicted parents’ their use of explicit mental state language (coded with the interactional mind-mindedness coding scheme) during interaction with their 7-month-old infants. The authors argue that parents’ use of mental state language during interaction is likely to mediate the association between parental offline reflective functioning and child outcomes, and they call for further studies on this subject. Another study [65] failed to find associations between PRF (assessed with the PRFQ) and mental state language (assessed with the interactional mind-mindedness coding scheme [60]). While this inconsistency between findings might be explained in terms of methodological differences (interview vs. questionnaire), it is also possible that a parent’s offline mentalizing ability does not necessarily translate into ‘mentalizing in action’, which may have different implications for the child. In the current study, we contribute to the mentalizing literature by examining the associations between offline self-reported PRF and parental mentalizing, assessed as mental state language during parent-child interactions, in relation to children’s pretend play complexity.

### Empirical links between parental mentalizing and child pretend play

Despite both theoretical and empirical associations between parental mentalizing and child symbolic representations (e.g., [24, 66, 67]), studies investigating the association between parental mentalizing and child pretend play are scarce. Indeed, to our knowledge, only three previous studies [12, 42, 68] have specifically investigated links between parental mentalizing and child pretend play. All three studies assessed child pretend play during interactions with a play partner (experimenter or parent).

In a sample of 206 mothers and their children, Meins and colleagues [42] found that less optimal mental state language during mother-infant interactions at eight months (assessed with the mind-mindedness coding scheme [60]) predicted lower levels of child pretend play during an instructed pretend play task at two years. Osório and colleagues [68] applied another measure to assess mothers’ use of mental state language (Mental State Talk, using coding procedures described in [69]) during joint pretend play with their 3-year-old children (N = 49). In this study, no association between maternal mental state talk and child pretend play in a separate task was found. Instead, mothers’ mental state talk was positively associated with their children’s use of mental state talk during joint pretend play, which in turn predicted children’s pretend play complexity. In a more recent study, Giovanelli and colleagues [12] used the mind-mindedness coding scheme to assess mothers’ use of mental state language during mother-infant pretend play at infant ages 6 and 12 months and infant symbolic representational abilities at ages 12 and 18 months. The results showed that mothers’ appropriate use of mental state language at 6 months was positively correlated with the infants’ level and duration of pretend play at 12 months. In accordance with theoretical assumptions [27, 39, 54], the authors argue that their findings support the idea that the caregiver’s online mentalizing abilities (referred to as attuned interpretations and expressions of their infants’ internal states) are predictive of the child’s emerging capacity for pretend play.

### Current study

In summary, there is some evidence that caregiver mentalizing facilitates children’s *social* pretend play. However, to our knowledge, no previous study has investigated links between parental mentalizing and child *solitary* pretend play. This is relevant because it can be argued that a child’s ability to pretend play without assistance from a parent or another player may index the child’s ‘baseline’ pretend play capacity, and as such, the child’s emerging symbolic capacity. Therefore, the overall objective of this study is to examine whether the capacity in 4-year-old children for solitary pretend play (operationalized as pretend play complexity) is associated with their mother’s mentalizing abilities.

While the three existing studies concerning parental mentalizing and child social pretend play [12, 42, 68] all examined online mentalizing, no studies have yet investigated offline mentalizing as a predictor of children’s solitary (or social) pretend play abilities. As there may be both unique and interrelated pathways from parental offline mentalizing and parental online mentalizing to child pretend play abilities, in this study, we add to the existing literature on parental mentalizing by investigating the association between caregiver offline reflective functioning and online mental state language [51, 52, 64, 65]. Because mental state language is a type of parenting behavior that more proximally shapes the child’s immediate experience, it is possible that this aspect of the parent-child interaction mediates the association between child outcomes and a more underlying, global ability to mentalize in parents. Hence, we investigate parental online mental state language as a mechanism through which offline parental reflective functioning facilitates the child’s capacity for solitary pretend play. Specifically, we hypothesize that an association between PRF and child solitary pretend play complexity is mediated by parental mental state language during interaction, with more optimal PRF being associated with more mental state language and more child pretend play complexity.

## Methods

### Procedure

This study was part of a broader investigation focusing on children’s play and caregiver-child interaction. The research received approval from the Institutional Ethical Review Board of the Department of Psychology at the University of Copenhagen (Approval number: 2015/11). We recruited mother-child dyads from parenting groups in and around Copenhagen, Denmark, primarily through social media channels. The inclusion criteria for the study were as follows: the child had to be the first-born, both mother and child needed to be Danish-speaking, and the child was required to be typically developing, aged between 4 years 0 months and 4 years 11 months.

Interested parents, after initiating contact with the research team, received detailed information about the study via email. Subsequently, they were invited, along with their child, for a two-hour session at the research unit’s laboratory. Upon their arrival, parents were provided with a comprehensive overview of the study, following which they provided their written consent. The laboratory visit was divided into three distinct parts: (1) a play session involving the mother and child, (2) an assessment session led by a researcher to evaluate the child’s verbal abilities, and (3) a questionnaire session, during which a research assistant administered a series of questionnaires to the mother. Parts 2 and 3 occurred concurrently, i.e., while the researcher tested the child, the mother was engaged in completing questionnaires in a separate room. The set of questionnaires included a socio-demographic survey that captured information such as maternal education level, age, and child gender, in addition to the Parental Reflective Functioning Questionnaire (PRFQ)–further details can be found in the Measures section. The sequence of the three parts were planned with the play session preceding the test and questionnaire sessions. This was chosen mitigate any potential influence that the latter sessions might have on the behavior of the child or the mother during the play session. The play session (part 1) consisted of seven consecutive episodes, each lasting five minutes. However, as the current study only utilizes data from the first two episodes, the subsequent episodes are not described in this paper. For a comprehensive description of all play episodes, readers are referred to Stuart et al., 2023 [70].

The play session took place in a dedicated room and was monitored by researchers through a one-way mirror and recorded via cameras installed in the lab capturing different angles of the room. The room was set up with a chair for the mother to sit on and magazines for her to read in. On the floor, a selection of LEGO DUPLO™ was provided, including a playhouse with furniture (four chairs, a table, a kitchen, two beds, and a toilet); figures (a man, a woman, a boy, a girl, a police officer and thief, and a princess and two knights); animals (a cow, a horse, and a crocodile); a motorcycle; a fire truck; 24 loose bricks; a slide; money; cake; a suitcase; and a sun/rain brick. The mother was instructed beforehand and given the opportunity to ask questions. The researcher signaled the beginning of each episode by knocking on the one-way mirror, and subsequently, the mother opened a corresponding envelope (e.g., envelope number two for episode number two) containing a short and simple instruction to the mother. The sequence of the play episodes was fixed rather than randomized to avoid a spill-over effect of the joint play session on the child’s solitary pretend play complexity.

#### Play episode 1: Child solitary play (5 min.)

The mother and child were introduced to the room and the instruction given was: “Please sit back, read a magazine, and let your child play with the toys”. Although this episode was designed to assess the child’s solitary pretend play complexity, the mother was present in the room with the child to facilitate a sense of safety allowing the child to explore the toys and focus on playing.

#### Play episode 2: Mother and child free play (5 min.)

The mother was invited to engage in free play with her child. The instruction provided was: “Please engage in free play with your child. Just do what you always do or play as you normally play with your child”. This episode aimed to assess maternal use of mental state language during play with her child.

### Measures

#### Pretend play complexity

To measure the complexity of child solitary pretend play, we used a global and integrated coding scheme that assesses both the quantity and the quality (i.e., the complexity of the level of pretend play) based on previous research [32, 71]. Belsky and Most’s 12-Step Play Scale [32] which includes definitions of functional play, levels of pretend play, substitutions, and sequenced play, and was utilized in this study. However, this scale does not encompass thematic complexity or role-play, so these aspects were supplemented from Shim (2007) [71]. Table 1 shows the pretend play complexity scale as used in the present study.

**Table 1:** Sample characteristics.

	M	SD	Range
Child age (years.months)	4.5	0.11	4.3–4.9
Maternal age (years)	34	4.28	24–44
Maternal length of education (years)	15.4	1.61	9–20
Child solitary play complexity	4.95	2.59	0–16
Maternal Online Mentalizing (PRFQ-IC^a^ scores)	6.25	0.59	4.3–7.0
Maternal Online Mentalizing (Mental State Language)	5.51	7.85	0–39
Child Verbal Comprehension (WPPSI-IV^b^)	113	19.42	54–141

^a^PRFQ-IC = Parental Reflective Functioning Questionnaire, Interest and Curiosity subscale

^b^WPPSI™-IV = Wechsler Preschool and Primary Scale of Intelligence™ –Fourth Edition

Simple, moderate, and advanced pretend play can all occur within a five-minute play session; therefore each child’s play is given a score between 0 and 3 within each category. A score of 0 indicates that the play behavior is not present at all. A score of 1 indicates that the play behavior is present for less than 1 minute out of 5. A score of 2 indicates that the play behavior is present 1–3 minutes. A score of 3 indicates that the play behavior is present for more than 3 minutes. The scores from the scales are then weighted and summed up for a final total score: (functional x 1) + (simple pretend x 2) + (moderate pretend x 3) + (advanced pretend x 5). Advanced pretend is multiplied by 5 to ensure that a score of 3 at level 4 (maximum score) is always the highest, giving extra weight to this level which surpasses the others. Otherwise, a child who plays at the moderate level for the maximum amount of time (yielding a final score of 9) could outscore a child who plays at the advanced level for 1–3 minutes (yielding a final score of 8). By weighting the scoring by 5, we ensure that a child engaging in advanced pretend play for at least 1 minute always achieves the highest score.

A research assistant, trained to reliability, coded each play session on a global scale. Complexity was rated on a four-point scale (0–3) according to the four categories, based on the degree of pretend complexity. Each child received a global score ranging from 0 to 16, representing their level of pretend play complexity. A second trained and reliable coder, blind to the primary coder’s scores, coded a randomly selected 20% (*n* = 21) of the data to determine interrater reliability. Interrater reliability was calculated using the average measures intraclass correlation coefficient (ICC) for the total scale, employing a two-way mixed-effects model with absolute agreement. The ICC was .88 (95% CI [.83; .91], p < .001), indicating good interrater reliability.

#### Parental reflective functioning

To assess offline parental mentalizing, operationalized as parental reflective functioning (PRF), we used the Parental Reflective Functioning Questionnaire (PRFQ: [41]. This is an 18-item self-report questionnaire intended for use with parents of children aged 0–5 years. Each item is rated on a 7-point Likert scale with “1” representing “strongly disagree” and “7” representing “strongly agree”. The PRFQ yields three subscales: (1) interest and curiosity in the child’s mental states, (2) certainty of the mental states of the child, and (3) prementalizing. This three-subscale structure holds for mothers and fathers and has been replicated in different independent samples (e.g., [72–74]), and across samples, the PRFQ has shown acceptable psychometric properties, including test-retest reliability over time and acceptable criterion reliability [58, 75].

Although each of the three scales represents unique aspects of parental reflective functioning, the ability to demonstrate genuine interest and curiosity in the internal world of the child is considered the hallmark of adaptive parental reflective functioning [41, 56, 76]. The Interest and Curiosity (IC) scale captures parents’ inclination to guess and understand what their child is thinking and feeling. It is well-suited to be utilized as a measure of parents’ general mentalizing capacities in non-clinical samples. Previous research has found that this subscale is negatively associated with lowered maternal emotional awareness [77] and positively associated with parental satisfaction, involvement with the child, and communication with the child [78]. Hence, we used the PRCQ-IC scale to assess maternal offline mentalizing. Cronbach’s α for the IC scale was .61, in the present sample which is comparable to what has been found in a similar sample [73] and considered acceptable [79].

#### Mental state language

The mothers’ use of mental state language during Play episode 2 (mother and child free play) was utilized as an indicator of their online mentalizing ability. To allow for as much naturally occurring mental state language as possible, and to avoid that the mental state language was inflated by the instruction, no instructions were given to the mother other than to play as she would normally do. To assess mental state language, we coded interactions with a modified version of the mind-mindedness coding scheme for interactional mind-mindedness [60] where only comments referring to internal states (referred to as ‘mind-related comments’ in the coding manual) were coded. The mind-mindedness measure [60] has been adapted in previous studies to assess mental state language during parent-child play (e.g., [80]), and we adapted the coding scheme to include maternal mental state language referring to a) the child´s mental states, b) the mother’s mental states, and c) mother speaking on behalf of the characters/figures in the play situation. We coded all maternal comments related to mental states concerning herself, the child, or the play figures and categorized them into one of the following three, mutually exclusive, categories: (1) “Desires and interest” (e.g., like/dislike, prefer, love/hate, want); (2) “Cognitions” (e.g., think, decide, know, remember, recognize, expect); (3) “Emotions” (e.g., happy, sad, scared, shy, confused, excited, upset). When coding for mental state language on behalf of the play figures, all of the mother’s utterances that were clearly intended to be said or thought on behalf of the play figures were coded in accordance with the coding criteria described in the mind-mindedness coding manual [60]. To index mental state language, both frequencies and proportions of mental state language have been coded in relation to the total number of comments uttered during the assessment, and studies report identical patterns regardless of the method applied [42, 81]. In this study, we used frequencies to index the mothers’ use of mental state language. A trained research assistant coded mental state language. To determine interrater reliability, we randomly selected 20% of the interactions that were rated by another trained coder blind to the primary coder’s ratings. The average measures intraclass correlation coefficients (ICC) for the total scale, using a two‐way mixed‐effects model with absolute agreement were ICC = .994 (95% CI [.992; .995], *p* < .001), indicating excellent interrater reliability.

#### Child language

Child verbal ability was assessed with Wechsler Preschool and Primary Scale of Intelligence™ –Fourth Edition (WPPSI™-IV [82]. Verbal IQ scores, known as child verbal comprehension index (VCI), were used in the analyses as a covariate, as child verbal IQ is known to correlate with the capacity for pretend play (for a meta-analysis, see [4]).

### Statistical analyses

All analyses were performed using SPSS Statistics 28 (IBM, Chicago, IL). A visual inspection determined the assumptions of normality and linearity approximately met, and the data did not show any problems of multicollinearity. Means, standard deviations, and ranges were computed to examine the degree of variation in the predictor (maternal offline mentalization), mediator (maternal online mentalization), and outcome (child solitary pretend play). Spearman correlations were conducted to assess how the variables were associated. To test our hypothesis, a mediation analysis was performed using the PROCESS macro for SPSS [83] that estimates the indirect effect, i.e., the combined effect of the predictor and mediator on the outcome. To estimate the indirect effect in the mediation analysis, we used bootstrapped bias-corrected and accelerated (BCa) confidence intervals (CI) based on 5,000 bootstrapped samples. If the null hypothesis (i.e. zero) was not included in the CI, it was interpreted as significant. Child VCI was controlled for in the mediation analysis, as child verbal IQ is known to correlate with the capacity for pretend play. All reported *p*-values are two-tailed and were evaluated at a 5% significance level.

## Results

The study initially enrolled 104 mothers. However, due to the unavailability of data for two children on the verbal IQ test and for three mothers on mental state language scores during interaction, these respective dyads were excluded from further analysis. Consequently, the final sample size for this study was reduced to *N* = 99.

### Descriptive statistics

Sample characteristics are presented in Table 1. As indicated, the mothers were generally well-educated with an educational level corresponding to ISCED level 5 and 6 and the first or second stage of tertiary education. Female children comprised 44.2% (*n* = 46) of the total sample, and the children scored close to one standard deviation above the mean on the VCI. While the children’s average score was in the lower range of the play complexity scale during solitary play (*M* = 4.95; *SD* = 2.59; range 1–16), the mothers’ average scores on the PRFQ-IC were in the high range of the scale (*M* = 6.25; *SD* = 0.59), and the number of maternal mental state language comments during joint play was on average 5.51 (*SD* = 7.85).

Table 2 presents the bivariate correlations between the study variables. Notably, only the child’s solitary pretend play complexity showed a significant correlation with the mother’s mental state language. This indicates that the more mental state language the mother used, the more complex the child’s solitary pretend play was.

**Table 2:** Bivariate correlations between the study variables.

	1.	2.	3.	4.
1. Child solidary play	-	.11	.27**	.14
2. Maternal Offline Mentalization (PRFQ-IC scores)		-	.14	-.01
3. Maternal online mentalization (Mental State Language)			-	.07
4. Child Verbal comprehension index (WPPSI-IV^b^)				-

**p < .01.

^a^PRFQ-IC = Parental Reflective Functioning Questionnaire, Interest and Curiosity subscale

^b^WPPSI™-IV = Wechsler Preschool and Primary Scale of Intelligence™ –Fourth Edition

### The relationship between parental reflective functioning and mental state language

The first model examined the effect of maternal (offline) PRFQ-IC on maternal (online) mental state language. Maternal PRFQ-IC scores significantly predicted maternal mental state language (*b* = 3.44, t(96) = 2.69, *p* = .008, 95% CI [0.91; 5.98]), with every 1 point increase in PRFQ-IC scores, she would increase 3.44 in her mental state language. This reflects that the more interested and curious the mother was about her child’s mental states, in general, the more she would display mental state language during joint play with her child. The first model explained 8% of the variance in (online) mental state language (F(2, 96) = 4.41, *p* = .015). This indicates that maternal offline mentalizing explains a small-to-medium effect [84] of the variance in maternal online mentalizing.

### The relationship between maternal mentalizing and child solitary play

The second model examined the effect of maternal mental state language and maternal PRFQ-IC on child solitary pretend play complexity. Here, there was a significant effect of maternal online mental state language on child solitary pretend play complexity (*b* = 0.11, *t*(95) = 3.23, *p* = .002, 95% CI [0.04; 0.17]). Specifically, for every 1 word increase in the mother’s online mentalizing (i.e., mental state language), her child’s pretend play complexity increased by 0.11 points. This suggests that a higher frequency of mental state language was related to more complexity in the child’s solitary pretend play. However, no significant direct effect was found for maternal PRFQ-IC scores on child solitary play complexity (*b* = 0.35, t(95) = 0.81, *p* = .42, 95% CI [-0.50; 1.20]). This may indicate that the mother’s interest and curiosity in her child’s mental states, as measured by PRFQ-IC, is not directly associated with pretend play complexity. The second model accounted for 15% of the variance in child pretend play complexity (F(3, 95) = 5.80, *p* = .001), suggesting that maternal offline and online mentalizing explains a moderate effect of the variance in the complexity of the child’s solitary pretend play.

### Mediation model

**Fig 1:**
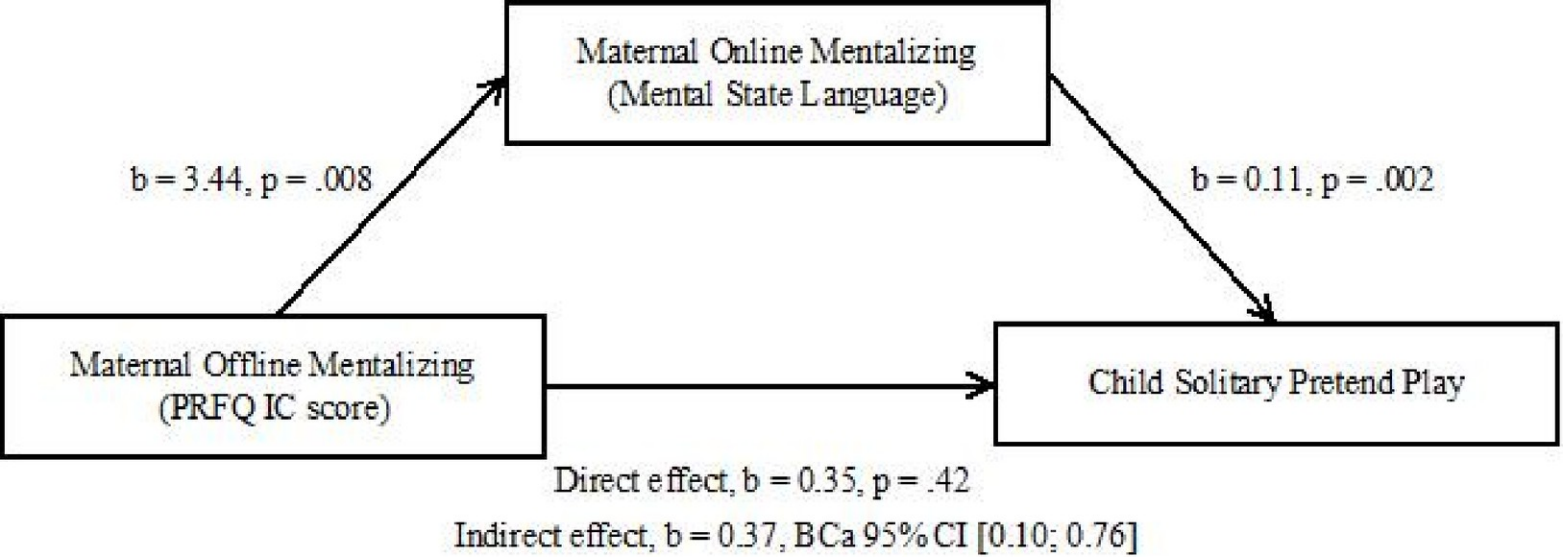
Model of maternal offline mentalization as a predictor of child solitary pretend play complexity, mediated by maternal online mentalization. The confidence interval for the indirect effect is a BCa bootstrapped CI based on 5000 samples.

Finally, we observed a significant indirect effect of the PRFQ-IC score on child solitary pretend play complexity, mediated through online maternal mental state language (*b* = 0.37, BCa 95% CI [0.10; 0.76]). To facilitate interpretation of this finding, given that all variables were measured on different scales, we standardized the results. The completely standardized indirect effect was significant (*b* = .08, BCa 95% CI [.03; .16]). This reflects that with an increase in PRFQ-IC scores, there was a corresponding increase of .08 standard deviations in the child’s pretend play complexity, attributable to the indirect effect of maternal mental state language during interaction with the child. Fig 1 illustrates a model depicting maternal PRFQ-IC score as a predictor of child solitary pretend play complexity mediated by maternal mental state language during interaction.

## Discussion

In this study, we investigated maternal offline mentalizing (self-reported PRF), and online mentalizing (mental state language during interactions) as predictors of 4-year-olds’ capacity for solitary pretend play in the presence of their mother. Children’s pretend play constitutes a window into their emerging capacity for symbolic functioning [5] and is closely related with their overall well-being and the quality of their caregiving environment [21]. By examining pretend play as a distinct outcome, we build upon the perspective of Bergen, [2] who suggested to focus on understanding what conditions enable children to become ‘excellent pretense players’. This approach moves beyond the traditional view of pretend play merely as a predictor of other developmental areas, such as cognitive or language development [1, 4, 85]. While both theoretical frameworks and empirical evidence suggest links between a caregiver’s capacity to understand and interpret the child’s internal world (mentalizing ability) and various child psychosocial outcomes [46, 49], empirical research specifically linking parental mentalizing to children’s pretend play abilities remains limited [12, 42, 68]. Our study contributes to this area of research.

Consistent with our hypothesis, our findings indicate that maternal online mentalizing, specifically the use of mental state language during interaction, serves as a mediator in the relationship between offline PRF and children’s capacity for solitary pretend play. Although the mediation analysis showed no direct effect of PRF on child pretend play, it did reveal a positive indirect effect. Specifically, a higher PRFQ-IC score was associated with increased complexity in child solitary pretend play, and this association was mediated by higher levels of maternal mental state language during interactions with the child.

Overall, our findings are in line with the results from two previous longitudinal studies [12, 42]. The authors of these studies report that maternal mental state language during interaction at 6 and 8 months, respectively, is related to the child’s level of social pretend play at 12 months with the mother [12] and at two years with an experimenter [42]. Our study extends these findings in two significant ways. Firstly, we assessed children’s pretend play at an age where this ability is believed to peak in typically developing children, specifically between 4 and 5 years [8–10]. This allows us to capture pretend play when children are likely to exhibit a greater range of complexity in their pretend play compared to earlier [13, 31]. Secondly, by focusing on child solitary (as opposed to social) pretend play, we may better capture the child’s ‘baseline’ pretend play ability, because the child’s pretend play complexity in our study is not facilitated or inflated by the mother’s or experimenter’s active participation in the play sequence.

In contrast to our results, Osorio and colleagues [68] found no main effect of online mentalizing, i.e., mental state language, on 3-year-old children’s social pretend play in a separate instructed play task with an experimenter. They suggested that maternal mentalizing might have played a more crucial role in the development of children’s pretend play at an earlier age. However, as the level of play quality was only credited if the children were able to incorporate an experimenter’s play suggestions into their play in this study, it raises questions about whether the task truly assessed the children’s core competence in pretend play. It might have been more indicative of the children’s collaborative skills rather than their pretend play abilities. Moreover, Osorio and colleagues measured maternal mental state language in a separate test specifically designed to elicit mental state language. Such tasks, designed to elicit mental state language, are known to potentially inflate and distort data [61]. Therefore, it could be argued that the naturally occurring mental state language assessed during joint play in our study, offers a more ecologically valid representation of the maternal online mentalizing abilities that are crucial for children’s pretend play ability.

Our study also extends previous research by investigating the links between offline PRF, as measured by the PRFQ, and online use of mental state language. It has been suggested that a parent’s use of mental state language is an expression of an underlying reflective capacity [51, 52], and while some studies have found associations between offline and online measures of mentalization, these tend to be small-to-moderate in size. Further, studies have used different methods for examining offline and online mentalizing, capturing different aspects of the construct of parental mentalizing [86]. In line with the findings of Rosenblum et al. [64] but contrasting those of Krink and Ramsauer [65], our results indicate a relationship between offline PRF and maternal mental state language during interaction. PRFQ-IC accounts for 8% of the variance in maternal mental state language which can be interpreted as a weak-to-medium association between these two constructs [84]. This suggests that the two constructs are interrelated but also capture different aspects of caregiver mentalizing abilities.

When comparing our findings with the previous studies, it is also important to consider the methodological differences. We used a questionnaire to assess PRF, whereas the study by Rosenblum and colleagues utilized an interview approach–specifically, the Working Model of the Child Interview [87] coded with the Reflective Functioning Scale [88]–which is generally considered the ‘gold standard’ for measuring mentalizing capacity [89, 90]. In line with their results, we found the expected association between the PRFQ-IC and mental state language. Interestingly, Krink and Ramsauer, who similarly to our approach also used the PRFQ to examine the links between offline PRF and online mental state language, found no associations between the PRFQ and maternal mental state language [65]. One possible explanation for the contrasting findings may pertain to differences between samples. Our sample consisted of non-clinical well-resourced mothers and their children, while the sample used in the study Krink and Ramsauer [65] was a clinical sample of mothers with severe postnatal mental illness. It has been suggested that associations between different dimensions of parental mentalizing may be influenced by parental psychological functioning [91–93]. It is possible that the so-called “competence-performance” gap [94] between ‘having’ the capacity to mentalize and ‘using it in action’, is less easily overcome when struggling with mental illness, as compared to our non-clinical sample. It should also be noted that a different subscale of the PRFQ other than the IC scale was used in the Krink and Ramsauer study [65]—specifically, the prementalizing subscale. The reason for this was that the prementalizing subscale is suggested to be relevant to postnatal psychopathology as it captures a more distorted and maladaptive non-mentalizing stance [41, 95]. We used the IC scale, as it is designed to capture individual differences in adaptive parental reflective functioning [41]. We therefore considered it suited for use in our typical sample where distorted parental mentalizing is expected to be less prominent. Yet, this difference in PRFQ subscales may provide an additional reason for the divergent results. In accordance with the conclusion of a recent review [86], we argue that future studies should examine how different aspects of PRF are associated with online mentalizing and child outcomes in both clinical and non-clinical parent-child dyads.

Our findings, that PRFQ-IC is only indirectly related to play complexity, may imply that PRF facilitates child development, and in this case, the child’s capacity for solitary pretend play, only if the caregiver can translate offline mentalizing into ‘mentalizing in action’. In other words, it is not merely how parents mentalize the child in general but how they verbally express an understanding of the child’s mental experience that is likely to promote the child’s ability to create a symbolic ‘as-if’ world where pretend play develops. The caregiver’s ability to show and actively express genuine interest and curiosity in mental states (their own, the child’s, and, in play, the pretended figures’) during day-to-day interactions is likely to enable a shared meaning-making to facilitate the understanding and representation of the child’s inner and external world.

### Limitations and future directions

Although we have exerted great effort to thoroughly test our hypothesis by including data on both offline and online mentalization, and by isolating the child’s capacity for pretend play from adult influences in the situation, this study has some limitations to consider. It could be argued that the temporal order of the measures is a limitation: Solitary play was assessed before both the mediator (assessed in Play Episode 2) and the predictor (assessed together with other questionnaires after the play session). The reason for this order was that during our pilot study of the lab visit, we found that conducting the test sessions first exhausted the children and diminished the quality of their play. Moreover, conducting the play session first allowed the children time to acclimate to the lab setting (with the mother present throughout) before being alone with the experimenter during the test situation. The rationale for assessing maternal mental state language after child solitary play was to avoid a potential spill-over effect of joint social play on child solitary play, as previously observed [96]. Since maternal reflective functioning is considered a relatively stable capacity [56], administering it after the play sessions was deemed optimal. Indeed, it is also possible that completing the PRFQ before assessing mental state language could prompt mothers’ attention to mental states, potentially inflating their online mentalizing abilities. In terms of capturing the effects of a global parental mentalizing ability on caregivers’ spontaneous use of mental state language in the specific interactional context with the child, this is not ideal. Nevertheless, with the design used in this study, we cannot rule out a potential effect of conducting the play sessions first.

Related to this, it is also important to consider the cross-sectional design in relation to testing mediation. While it is not possible to strictly assert causality between caregiver mentalizing and child pretend play, we contend that the analysis adds significant value to the field (cf. [97]). In the classic tri-factor mediation model, all possible orders of the variables fall within the same Markov equivalence class (meaning they share same covariance structure). Consequently, it is never possible to establish causal order through significance testing alone [98]. Establishing causality of the variables is only possible through research design (e.g., randomization) or roboust theoretical assumptions [98]. We have delineated the theoretical assumptions of our model and argue that our study lends support to our hypothesized direction of effects. Nevertheless, future research should vary the temporal order of the measures in more sophisticated research designs (e.g., longitudinal) to further investigate a potential causal effect of mentalizing on pretend play complexity.

Another limitation is the absence of a standardized test for pretend play. Pretend play ability is notoriously difficult to define and capture, and there are no universally accepted standards within the field [99]. However, there is a broad consensus regarding what constitutes pretend play, and what is considered more complex forms of pretend play. To capture individual differences in children’s capacity for pretend play, we used a previously applied play scale designed to measure varying degrees of complexity. The high levels of inter-rater relatability between coders reflect that it was a reliable measure. Nonetheless, the lack of a standardized measure, complicates the comparison of results across studies. Hence, future research focusing on how to measure the core elements and developmental progression of pretense in children are needed [99].

In our study, maternal mental state language was reflected by a singular score. This score derived from the mother’s references to mental states concerning the child, herself, as well as the play figures. Being a multifaceted construct, mentalizing is not only considered to be influenced by context (such as offline versus online assessment) but it has also been proposed that a distinction between self-focused and other-focused mentalizing should be made [75, 91]. Indeed, research on parental mentalizing has demonstrated distinct associations between parents’ self-focused and child-focused mentalizing and child outcomes [100–102]. Consequently, by merging references to mental states of various agents (self, child, play figures) into a single score, our study might have missed capturing how specific facets of online mentalizing language relate individually to child pretend play, potentially masking differing results. It should also be noted that while mentalizing includes verbally labeling mental states, mental state language is not inherently synonymous with mentalizing [52]. In this study, we operationalized parental online mentalizing in terms of mental state language during interaction with the child. However, further research is necessary to explore the construct validity of this method against other established measures of mentalizing.

All participants volunteered to participate in the study, and as such, it is possible that self-selection bias may have influenced our results. The sample was generally well-resourced, as indicated by the high levels of education and elevated PRFQ-IC scores among the mothers, as well as the children’s scores on the VCI. At the same time, this context may be beneficial for examining associations between the study variables, as risk factors potentially influencing child pretend play capacity and development (e.g., non-optimal caregiving and exposure to traumatic events) are likely to be very rare in this sample.

Finally, as most theories on the role of caregiver mentalization in child pretend play focus on maternal mentalization, our sample inclued only mother-child dyads. However, some researchers contend that fathers may have a more significant influence on children’s play and exploration than mothers do [103]. Indeed, the role of fathers in child play has not been as thoroughly researched as maternal influence. While we found that maternal mentalization positively predicts child pretend play, there are likely other mechanisms through which children develop the capacity for pretend play. Potential areas for future research could include paternal online mentalization, the influence of peers and siblings, or the role of professional caregivers.

## Conclusions

Overall, our study makes a significant contribution to the literature on how various conceptualizations and dimensions of parental mentalizing are related to child outcomes. Our findings empirically support theories that posit a link between parental mentalization and the child’s capacity for pretend play, specifically the notion [54, 104] that parents’ ability to understand and represent the child’s internal states can enhance capability for solitary pretend play. The discovery that offline PRF is only indirectly related to child pretend play complexity through online mental state language suggests that parental reflective functioning may only promote child mental development if the caregiver is able to effectively translate this capacity into ’mentalizing in action’ during interactions with the child.
